# Liver organoids reproduce alpha-1 antitrypsin deficiency-related liver disease

**DOI:** 10.1007/s12072-019-10007-y

**Published:** 2019-12-13

**Authors:** Gema Gómez-Mariano, Nerea Matamala, Selene Martínez, Iago Justo, Alberto Marcacuzco, Carlos Jimenez, Sara Monzón, Isabel Cuesta, Cristina Garfia, María Teresa Martínez, Meritxell Huch, Ignacio Pérez de Castro, Manuel Posada, Sabina Janciauskiene, Beatriz Martínez-Delgado

**Affiliations:** 1 Molecular Genetics Unit, Institute of Rare Diseases Research, Institute of Health Carlos III (ISCIII), Ctra. Majadahonda-Pozuelo Km2,200, 28220 Madrid, Spain; 2 General and Digestive Surgery Department, Hospital Doce de Octubre, Madrid, Spain; 3 Bioinformatics Unit, Institute of Health Carlos III (ISCIII), Madrid, Spain; 4 Digestive Department, Hospital Doce de Octubre, Madrid, Spain; 5 Neumology Department, Hospital Doce de Octubre, Madrid, Spain; 6 Wellcome Trust–Medical Research Council Stem Cell Institute, University of Cambridge, Cambridge, UK; 7 Gene Therapy Unit, Institute of Rare Diseases Research, Institute of Health Carlos III (ISCIII), Madrid, Spain; 8 Institute of Rare Diseases Research, Institute of Health Carlos III (ISCIII), Centre for Biomedical Network Research on Rare Diseases, CIBERER, Madrid, Spain; 9 Department of Respiratory Medicine, German Centre for Lung Research (DZL), Hannover Medical School, Hannover, Germany

**Keywords:** Organoids, Liver, Alpha-1 antitrypsin deficiency, Oncostatin M, RNA-seq, SERPINA1

## Abstract

**Background and aims:**

Alpha-1 antitrypsin (AAT) is a product of *SERPINA1* gene mainly expressed by hepatocytes. Clinically relevant mutations in the *SERPINA1* gene, such as Z (Glu342Lys), results in an expression of misfolded AAT protein having high propensity to polymerize, accumulate in hepatocytes and thus to enhance a risk for hepatocyte damage and subsequent liver disease. So far, the relationship between the Z-AAT accumulation and liver cell damage remains not completely understood. We present three-dimensional organoid culture systems, as a novel tool for modeling Z-AAT-related liver diseases.

**Methods:**

We have established liver organoids from liver biopsies of patients with homozygous (ZZ) and heterozygous (MZ) deficiency and normal (MM) genotypes of AAT. The features of these organoid models were characterized by analyzing AAT protein secretion and intracellular aggregation in MZ and ZZ genotypes as well as *SERPINA1* expression in differentiated cultures.

**Results:**

Transcriptional analysis of differentiated organoid cultures by RNA-*Seq* showed hepatocyte-specific gene expression profile. Genes, such as *ALB, APOB, CYP3A4* and *SERPINA1*, were validated and confirmed through quantitative-PCR analysis. The organoids from MZ and ZZ cases showed intracellular aggregation and lower secretion of AAT protein, and lower expression of *ALB* and *APOB*, as typically seen in hepatocytes from Z-AAT deficiency patients. Furthermore, organoids responded to external stimulus. Treatment with oncostatin M, a well-known inducer of *SERPINA1*, increased expression of the full-length transcripts (AAT-1C) as well as the short transcript of AAT (AAT-ST1C4).

**Conclusions:**

Liver organoid model recapitulates the key features of Z-AAT deficiency and provides a useful tool for disease modeling.

**Electronic supplementary material:**

The online version of this article (10.1007/s12072-019-10007-y) contains supplementary material, which is available to authorized users.

## Introduction

Alpha1-antitrypsin (AAT), a prototypic member of the serine protease inhibitor (serpin) superfamily, is mainly produced by hepatocytes. A normal concentration of the AAT in human blood ranges between 1 and 2 g/l and it is estimated that liver accounts for about 80% of the total circulating AAT [1]. Human AAT is also an acute phase glycoprotein with broad anti-protease and anti-inflammatory functions, and therefore AAT deficiency leads to various health problems, including liver disorders [2].

AAT is encoded by the *SERPINA1* gene located at the distal end of the long arm of chromosome 14. The typical *SERPINA1* gene consists of two alleles, named M, which are responsible for the synthesis of quantitatively and qualitatively normal AAT. The most frequent deficient alleles are so called S (Glu264Val) and Z (Glu342Lys). The combinations of the M, S and Z alleles give rise to the different genotypes MM, SS, MZ, SZ and ZZ. The homozygous ZZ genotype is the most relevant genotype in the clinical and genetic knowledge of *SERPINA1,* which results in about 90% reduced levels of circulating AAT protein. The deficiency in ZZ cases occurs due to the aberrant folding of the Z-AAT causing its polymerization and intracellular accumulation. The clinical manifestations of severe AAT deficiency include liver (intracellular retention of aggregated AAT that resists degradation) and lung (missing protective levels of functional AAT) diseases, and less frequently skin diseases such as panniculitis or ANCA + vasculitis [3].

The AAT deficiency-related liver damage can occur at any age. Clinical studies have shown that children who progressed to the end-stage liver disease had more severe abnormalities in infancy such as persistent jaundice for more than 6 weeks, hepatomegaly, higher transaminases and severe morphological changes including bile duct reduplication, fibrosis and cirrhosis. Currently, however, there are no distinguishable features/markers allowing to predict which child will develop a fast decline in liver function requiring liver transplantation or who will recover without sequelae of chronic liver disease [4]. In adults, liver damage can be manifested by liver fibrosis and cirrhosis, and hepatocellular carcinoma [3, 5]. On the other hand, Z-AAT deficiency carriers may remain clinically healthy until later adulthood. This variability in clinical presentation suggests that in addition to inherited abnormality in AAT protein, other environmental, genetic and epigenetic factors are necessary to promote the development of the AAT deficiency-related liver disease. Therefore, better understanding of the molecular mechanisms underlying liver disease related to Z-AAT deficiency is of critical importance for the diagnosis and the development of specific and personalized therapies.

Currently, experimental studies investigating liver disease in AAT deficiency are limited by the difficulty to obtain human liver tissue and to maintain primary cultures of human hepatocytes. Alternatively, human embryonic stem cells and induced pluripotent stem cells are used [6]. However, full differentiation of stem cells into mature hepatocytes has yet not been reported.

Organoids are new three-dimensional (3D) model systems referred to a group of cells growing in a 3D structure that are generated from primary tissues or cells, with self-renewal and self-organization capacity, maintaining similar appearance and functionality as the original tissue. Adult tissue-derived organoids can be maintained through indefinite passage and preserve genetic stability [7]. Recently, human liver organoids started to be used for the studies of various liver diseases [8, 9]. The first described human liver organoids allowed the expansion of adult liver stem cells and subsequent differentiation to hepatocytes that recapitulate some function of ex vivo liver tissue. Moreover, differentiated liver organoids from AAT-deficient patients mimicked the characteristics of the disease [7]. In this study, we have established and compared adult human liver organoids from liver biopsies of individuals with normal, MM and deficient ZZ and MZ AAT genotypes. The aim was to show if liver organoid cultures can recapitulate the typical features of liver cells expressing normal and deficient AAT and can be useful for AAT deficiency-related liver disease modeling. Typical features of AAT deficiency-associated liver disease were analyzed in terms of AAT polymerization and secretion, and transcriptional induction of *SERPINA1* gene transcripts in organoids subjected to external stimuli. The results show that liver organoids is a useful tool allowing modeling liver disease in individuals with different AAT mutations.

## Materials and methods

### Patients and genotyping

Organoids were established from liver biopsies collected from patients and controls at the Hospital 12 de Octubre in Madrid (Spain) and also provided by Dr. Huch at Cambridge University (UK). The ZZ organoids were derived from ZZ AATD patients with hepatic failure who had liver transplant, whereas MZ organoids were obtained from an adult MZ AATD patient who underwent colicestomy. The control MM AAT organoids were derived from an individual with hepatocellular carcinoma undergoing surgical resection. Tissue sample was obtained from macroscopically defined non-neoplastic adjacent area. All biopsies were genotyped for *SERPINA1*. Sequencing of *SERPINA1* gene coding exons was performed by using previously described primers [10, 11] in an automatic sequencer (ABI PRISM 377 Applied BioSystems). Signed informed consent for the study was obtained from all the subjects and the research was approved by the ethics committee of Instituto de Salud Carlos III, Madrid, Spain.

### Establishment and culture of human liver organoids

**Fig. 1:**
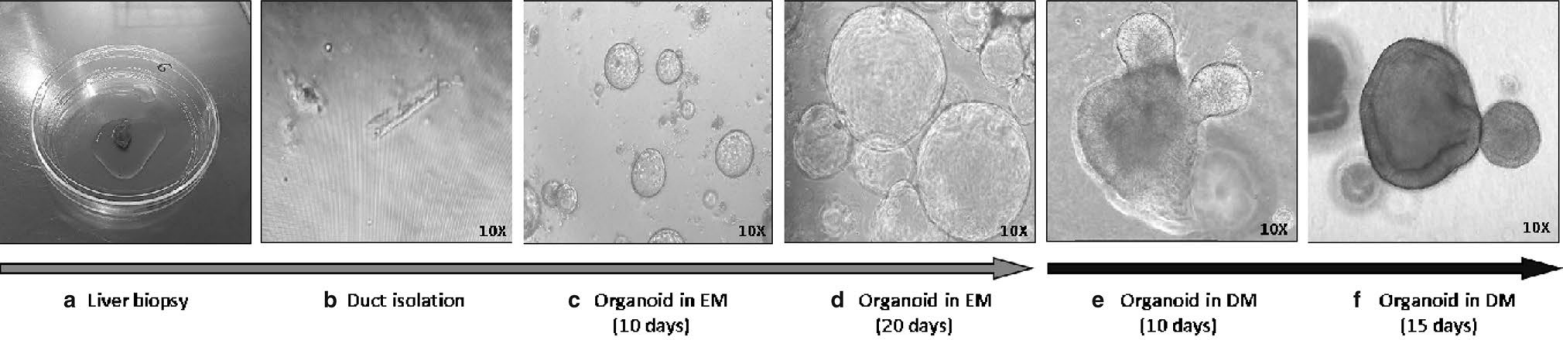
Representative images of the expansion and differentiation of liver organoids. **a** Human liver organoids were obtained from a liver biopsy of patients. **b** Ductal cells are isolated, **c**, **d** Liver organoids are developed by growing in an expansion medium (EM) and a 3D Matrigel, **e**, **f** Cultured in differentiation medium (DM) giving rise to differentiated organoids (magnification ×10). Microscope Leica DMIL LED, camera Leica MC170HD

We aimed to generate liver organoids expressing ZZ and MZ genotypes of AAT, and normal MM variant of AAT. Following the protocol described by Huch and collaborators [7, 12], we established liver organoids from ductal cells of human liver. Briefly, after surgical excision, the tissue was kept cold in basal medium (Advanced DMEM/F12, 1% penicillin/streptomycin, 1% Glutamax, 10 mM Hepes) until processing. The tissue was minced, washed with cold medium (DMEM, Glutamax, DGlu, Pyruvato, 1% FBS, 1% penicillin/streptomycin) and digested (Earle’s Balance Salt Solution (EBSS), collagenase D 2.5 mg/ml, DNAse I 0.1 mg/ml). After centrifugation, cells were resuspended in a basement matrix and seeded in a 24-well plate with isolation medium (basal medium supplemented with 25 ng/ml recombinant human Nogging, 30% conditioned medium with Wnt3a and 10 μM Rho kinase (Rock inhibitor)). In this specific culture condition, only progenitor adult stem cells, mainly located in ducts, are able to grow forming self-renewing organoids in the form of spheres. In each passage, organoids were removed from the basement matrix and transferred to fresh matrix. For expansion, the undifferentiated organoids were cultured in an expansion medium (EM) (basal medium, 1 mM *N*-acetylcysteine, 5% conditioned medium Rspo1, 10 mM nicotinamide, 10 nM recombinant human gastrin I, 50 ng/ml recombinant EGF, 100 ng/ml recombinant human fibroblast growth factor (FGF) 10, 50 ng/ml recombinant human hepatocyte growth factor (HGF), supplemented with 10 μM Rho kinase (Rock inhibitor)). The differentiation into hepatocytes was achieved with a differentiation medium (DM) (basal medium, 1:50 B27 supplement with or without vitamin A, 1:100 N2 supplement, 1 mM *N*-acetylcysteine, 10 nM recombinant human-gastrin I, 50 ng/ml recombinant mouse EGF, 100 ng/ml recombinant human FGF10, 50 nM A83-01, 10uM γ-secretase inhibitor (DAPT). Figure 1 illustrates different stages of the isolation, expansion and differentiation of the liver organoid.

### Identification of Z-AAT aggregates by using PAS-D staining

To detect intracellular aggregates of the Z-AAT protein, cytospins of organoid liver cells were stained with the periodic acid–Schiff–diastase (PAS-D). Organoids were washed with phosphate-buffered saline (PBS), spread on slides (1 min at 600 rpm) and fixed with 95% ethanol for 5 min. Afterward, cells were washed twice with PBS and stained with hematoxylin–eosin and PAS-D.

### Detection of intracellular AAT by immunofluorescence

Organoids growing in EM (undifferentiated) for 1–2 months or differentiated organoids growing in DM for 2 weeks were collected for immunofluorescence detection of total AAT or polymers of AAT. Cytospins of organoid cells were washed with PBS, fixed with 95% ethanol and permeabilized with 0.1% Triton X-100/PBS and blocked with 1% bovine serum albumin/phosphate-buffered saline (BSA/PBS). The expression of AAT protein was analyzed by incubating with anti-AAT B9 antibodies (sc-59438 Santa Cruz Biotechnology, Santa Cruz, CA, USA) for total AAT and with D11 antibody specific for AAT polymers (provided by Sabina Janciauskiene, Hannover). A secondary anti-mouse FICT IGG (Sigma F0257) antibody was used. 4′, 6-diamidino-2-phenylindole (DAPI Sigma) staining of cell nuclei was performed. The preparations were examined under a fluorescence microscope Zeiss Ax10. The proportion of positive cells showing AAT polymers (D11) was estimated by counting at least 300 nuclei from three different fields from MM, MZ and ZZ organoids (counting was performed by two independent researchers).

### Western blot analysis of AAT

Native and polymeric forms of AAT protein were detected in cell pellets and culture medium of expanding and differentiated organoids. Cell pellets were lysed with RIPA buffer containing a cocktail of complete protease inhibitors (Roche, Mannhein, Germany). The lysates and culture media were electrophoresed on 10% polyacrylamide sodium dodecyl sulfate PAGE (SDS-PAGE). The insoluble fraction was sonicated and separated on non-denaturing 8% PAGE. Proteins were detected with the following primary antibodies: anti-AAT B9 (1:100 dilution) and anti-beta-actin (AC-74 Sigma Aldrich, Taufkirchen, Germany) (1:5000 dilution) followed by chicken anti-mouse IgG-HRP (sc-2954 Santa Cruz Biotechnology) (1:5000 dilution), and visualized after labeling with Immobilon HRP substrate (Millipore) using Chemidoc Touch imaging system (Biorad, Hercules, CA, USA). Quantification of the western blot bands of AAT protein of MM, MZ and ZZ organoids was performed with Image J software.

### Transcriptomic analysis of liver organoids (RNA-Seq)

Total RNA was isolated from organoid cells using TriReagent (Sigma) followed by DNase1 digestion step to ensure that the samples were not contaminated with genomic DNA. The purity of RNA was assessed using Agilent RNA 6000 Nano Kit and the Agilent 2100 Bioanalyzer. TruSeq Stranded mRNA Kit (Illumina) was used for library preparation based on the recommendations from the manufacturer. The sequencing of the library was performed at the Genomics service (ISCIII) on a NextSeq 500 sequencer using 75 base read lengths in paired-end mode. The obtained RNA-*Seq* data were analyzed by the Bioinformatics Facility (ISCIII). First, quality control analysis involving fastQC v0.11.3 (http://www.bioinformatics.babraham.ac.uk/projects/fastqc/) was carried out, and any adapter sequences as well as low quality 3′ ends were removed using Trimmomatic v0.36. The high-quality reads were then mapped against Hg38 human genome using Tophat v2.0.14 and mapping quality control was performed using RseQC v2.6.4. Transcriptome prediction and gene/isoform quantification was calculated using Cufflinks v2.2.1 based on Hg38 RefSeq reference genes. Finally, differential expression analysis was carried out using cuffdiff. Differentially expressed genes (DEGs) were considered when FDR values were < 0.05. The CummeR bund R package (v2.14.0) was used for quality control and results visualization. The functional analysis was carried out with Functional Annotation Tool DAVID Bioinformatics Resources 6.8, NIAID/NIH.

### Validation of differentiation markers by RT-qPCR

Progenitor and ductal cell markers, leucine-rich repeat containing G protein-coupled receptor 5 (*LGR5)* and keratin 19 (*KRT19*), and differentiation markers (albumin, *ALB;* apolipoprotein B*, APOB;* and cytochrome P450 3A4, *CYP3A4*) were analyzed by RT-qPCR. Total RNA was extracted from organoids by using TriReagent (Sigma) and expression was also analyzed in HepG2 cells and control liver biopsy. cDNA was synthesized using Maxima First Strand cDNA Synthesis kit (Thermo Scientific, Fermentas Life Sciences, St. Leon-Rot, Germany). The RT-qPCR of selected hepatocyte markers was performed in triplicate using Taqman Fast Advance master mix (Applied BioSystems) and specific primers and probes (Supplementary Table 1). Taqman probes were from Universal probe library, UPL, (Roche): *KRT19* (#71), *LGR5* (#78), *ALB* (#44), *APOB* (#90), *CYP3A4* (#50). Amplification conditions were as follows: 95 °C for 20 s, 45 denaturation cycles at 95 °C for 3 s, annealing at 60° C for 30 s. RT-qPCR was performed on the QuantStudio 5 System (ThermoFisher Scientific) and the analysis using the software QuantStudio Design and Analysis Software v1.4.3. Glyceraldehyde-3-phosphate dehydrogenase (GADPH) was used as an endogenous control. The relative gene expression was calculated by comparative Ct method and obtaining the fold-change value (DDCt).

### Expression analysis of *SERPINA1* transcripts

The expression of alternative *SERPINA1* gene transcripts was analyzed in RNA from MM, MZ and ZZ organoids under both the expansion (EM) and differentiation (DM) conditions. The RT-qPCR for *SERPINA1* transcripts, generated by the use of different promoters and alternative splicing between non coding exons 1A, 1B and 1C, was performed with primers and methods previously described [13]. In addition, expression of *SERPINA1* short transcripts was analyzed as described [14].

### Exogenous organoid stimulation with OSM

To study the expression regulation of *SERPINA1* gene, organoids were exposed to oncostatin M (OSM: rhOncostatin M recombinant human *E. Coli* from RD Systems 295-OM). We added OSM (50 ng/ml) to organoids at day 14 of the differentiation process. After 16 h of stimulation, cells were collected and the expression of *SERPINA1* transcripts was quantified. A Student’s *t* test analysis was performed to compare the gene expression between controls and stimulated cells. Statistically significant values were considered if *p* < 0.05.

## Results

### Hepatocyte-specific gene expression in differentiated organoids

**Fig. 2:**
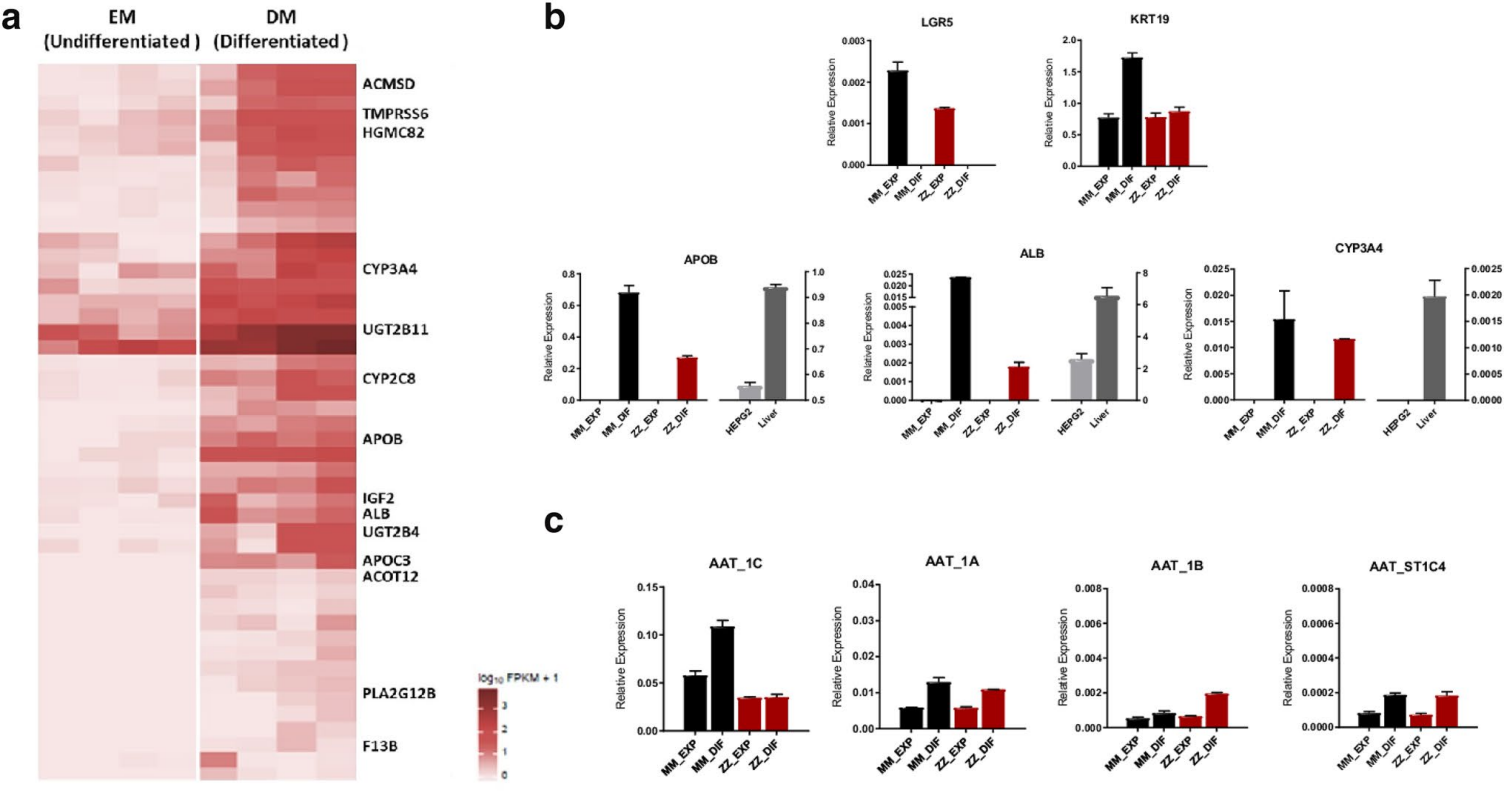
Gene expression analysis in human liver organoids. **a** Heatmap of the top up-regulated differentially expressed genes between undifferentiated organoids (EM) and organoids differentiated into hepatocytes (DM). Some of the most relevant mature hepatocyte genes are represented at the right side. Darker color means higher expression levels. Gene expression analysis of differentiation markers in organoids MM (black) and ZZ (red) under expansion and differentiation conditions: **b** expression of markers of pluripotent (*LGR5*), ductal cells (*KRT19*) and differentiated hepatocytes (*ALB*, *APOB* and *CYP3A4*); **c** expression of alternative transcripts of *SERPINA1* gene (1C, 1A and 1B) and short transcripts (ST1C4) in MM and ZZ organoids

Transcriptome analysis by RNA-*Seq* was used to compare undifferentiated and differentiated liver organoids independently of the genotypes. As expected, a high number of differentially expressed genes DEGs (3097 genes) were found between the undifferentiated and differentiated organoids. The top hundred most differentially expressed genes are illustrated in Fig. 2a, which include 48 up-regulated and 53 down-regulated genes after differentiation. Highly induced genes included typical mature hepatocyte genes, as previously described by other authors [15, 16]. The results confirmed that differentiated organoids, regardless of the AAT genotype, show increased expression of hepatocyte-specific genes such as albumin (*ALB),* apolipoprotein B (*APOB)*, apolipoprotein C3 (*APOC3*), cytochromes *CYP3A4, CYP2C8 (cytochrome P450 family 2 subfamily C member 8)*, as well as metabolism enzymes phospholipase A2 group XIIB (*PLA2G12B),* acyl-CoA thioesterase 12 *(ACOT12),* UDP glucuronosyltransferase family 2 member B4 *(UGT2B4),* insulin-like growth factor 2 receptor (*IGF2R*), UDP glucuronosyltransferase family 2 member B11(*UGT2B11*)*, 3*-*hydroxy*-*3*-*methylglutaryl*-*CoA synthase 2 (HMGCS2),* transmembrane serine protease 6 (TMPRSS6), aminocarboxymuconate semialdehyde decarboxylase *(ACMSD)* and coagulation factor XIII B chain *(F13B)*. A complete list of the top 100 most DEGs in differentiated organoids can be found in supplementary Tables 2 and 3.

The expression of certain progenitor, ductal and hepatocyte cell markers were validated in MM and ZZ by RT-qPCR (Fig. 2b). As expected, both MM and ZZ organoids when growing in EM expressed markers of progenitor (*LGR5*) and ductal (*KRT19*) cell lineage, but these markers disappeared after differentiation. In contrast, organoids cultured in DM highly expressed hepatocyte genes, such as albumin (*ALB*), apolipoprotein B (*APOB*) and cytochrome P450 3A4 (*CYP3A4*), which were mostly not detected in undifferentiated liver organoid cultures. Remarkably, when compared to MM AAT organoids, lower expression of the *APOB* and *ALB* hepatocyte markers, but increased expression of the *CYP3A4*, was observed in ZZ AAT organoids.

### SERPINA1 gene transcripts expression in liver organoids under expansion (EM) and differentiation (DM) conditions

The expression of full-length and short (*ST1C4*) transcripts of *SERPINA1* gene [14] was analyzed in MM and ZZ AAT-derived liver organoids (Fig. 2c). Higher expression of all *SERPINA1* transcripts was observed in differentiated than in undifferentiated MM and ZZ organoids, indicating that *SERPINA1* gene is induced with the differentiation process in hepatocytes. Moreover, *SERPINA1* expression levels were higher in MM than in ZZ differentiated hepatocytes. Interestingly, in ZZ AAT organoids the expression of 1C transcript, the main liver transcript, did not increase during the differentiation process (Fig. 2c). Only a slight increase in the expression of the other minor AAT transcripts originated by the monocyte promoters (1A and 1B) was detected. A short *ST1C4* transcript of *SERPINA1* gene was induced in differentiated M and Z hepatocytes as compared to undifferentiated organoids.

### Regulation of SERPINA1 gene transcripts by oncostatin M (OSM)

**Fig. 3:**
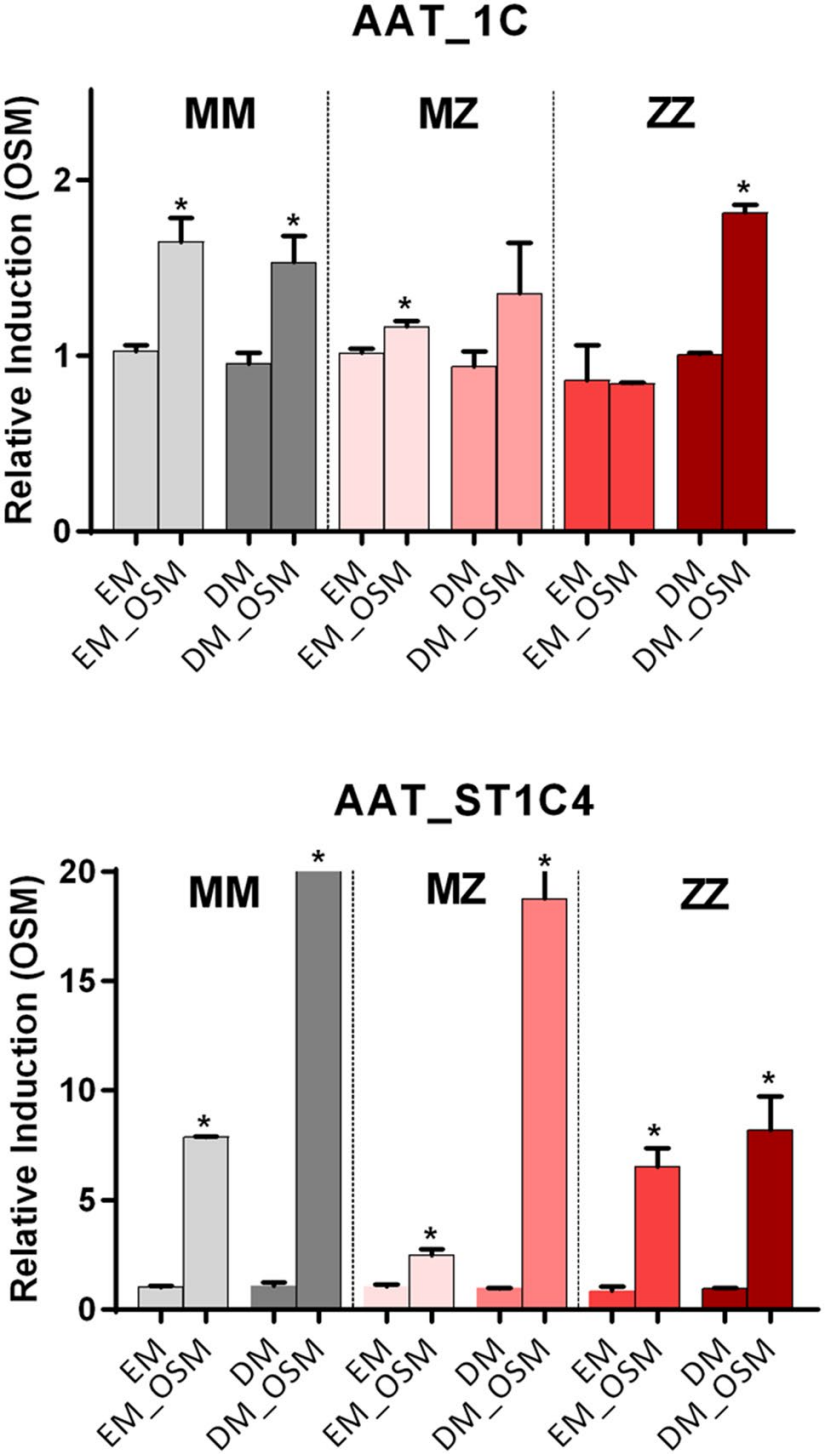
Induction of *SERPINA1* gene transcripts (1C) and short transcripts (ST1C4) by stimulations with OSM. Relative gene expression of the analyzed transcripts was measured in organoids grown in expansion medium (EM) and in differentiated organoids (DM). Statistically significant values are shown by * (*p* < 0.05)

The production of AAT in hepatocytes is controlled by a variety of cytokines released during inflammation, whereas leading regulators are IL-6-type cytokines. Therefore, we next analyzed transcriptional regulation of *SERPINA1* gene in liver organoids treated with oncostatin M, a pleiotropic cytokine that belongs to the IL-6 group of cytokines, a well-known inducer of AAT production [17]. This analysis was performed on both undifferentiated (EM) and differentiated (DM) organoids from MM, MZ and ZZ AAT cases (Fig. 3). In MM AAT organoids, the 1C full-length transcript of AAT, which is typically expressed by hepatocytes, was induced by OSM under both EM and DM conditions. However, in ZZ organoids, the more evident induction of 1C transcript was observed in the differentiated stage. It is important to point out that in differentiated hepatocytes, OSM also induced the expression of transcripts derived from the monocyte promoter 1A and 1B (Supplementary Fig). Short transcripts ST1C4 were highly induced by OSM in differentiated MM and MZ AAT organoids, and to a lesser extent in ZZ AAT organoids (Fig. 3). No expression of the other described short transcript, ST1C5, was detected in the liver organoids.

### Detection of PAS-D-positive AAT polymers in liver organoids

**Fig. 4:**
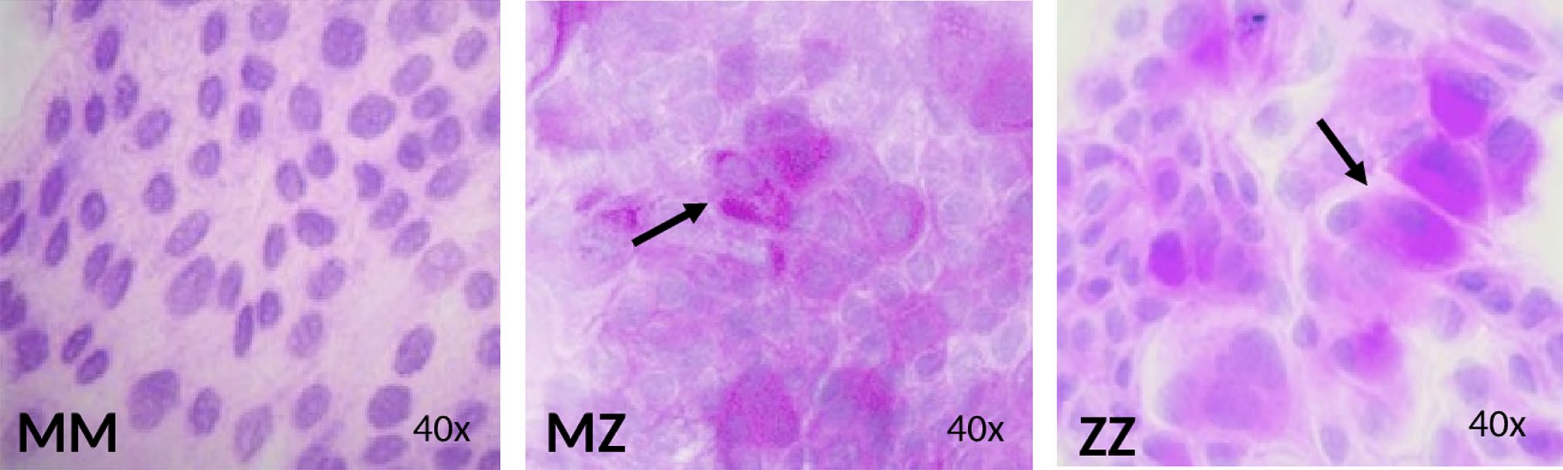
PAS-D staining for the detection of Z-AAT protein aggregates. Differentiated hepatocytes from MM organoids did not show PAS+ staining, while the MZ and ZZ organoids show evident aggregates of Z polymers. Microscope Leica DM 2000, camera Leica DFC450 and software LAS v4 (magnification ×40)

The presence of aberrant AAT polymers positive for PAS diastase-resistant (PAS-D) staining within hepatocytes is a characteristic feature of Z-AAT-deficient liver [3, 5, 18]. As expected, positive PAS-D staining was observed in organoids generated from the ZZ and from MZ AAT patients indicating the accumulation of the Z-AAT polymers within differentiated organoid cells. By contrast, PAS-D staining was negative in MM AAT organoids (Fig. 4).

### Detection of total and polymeric form of AAT protein in liver organoids

**Fig. 5:**
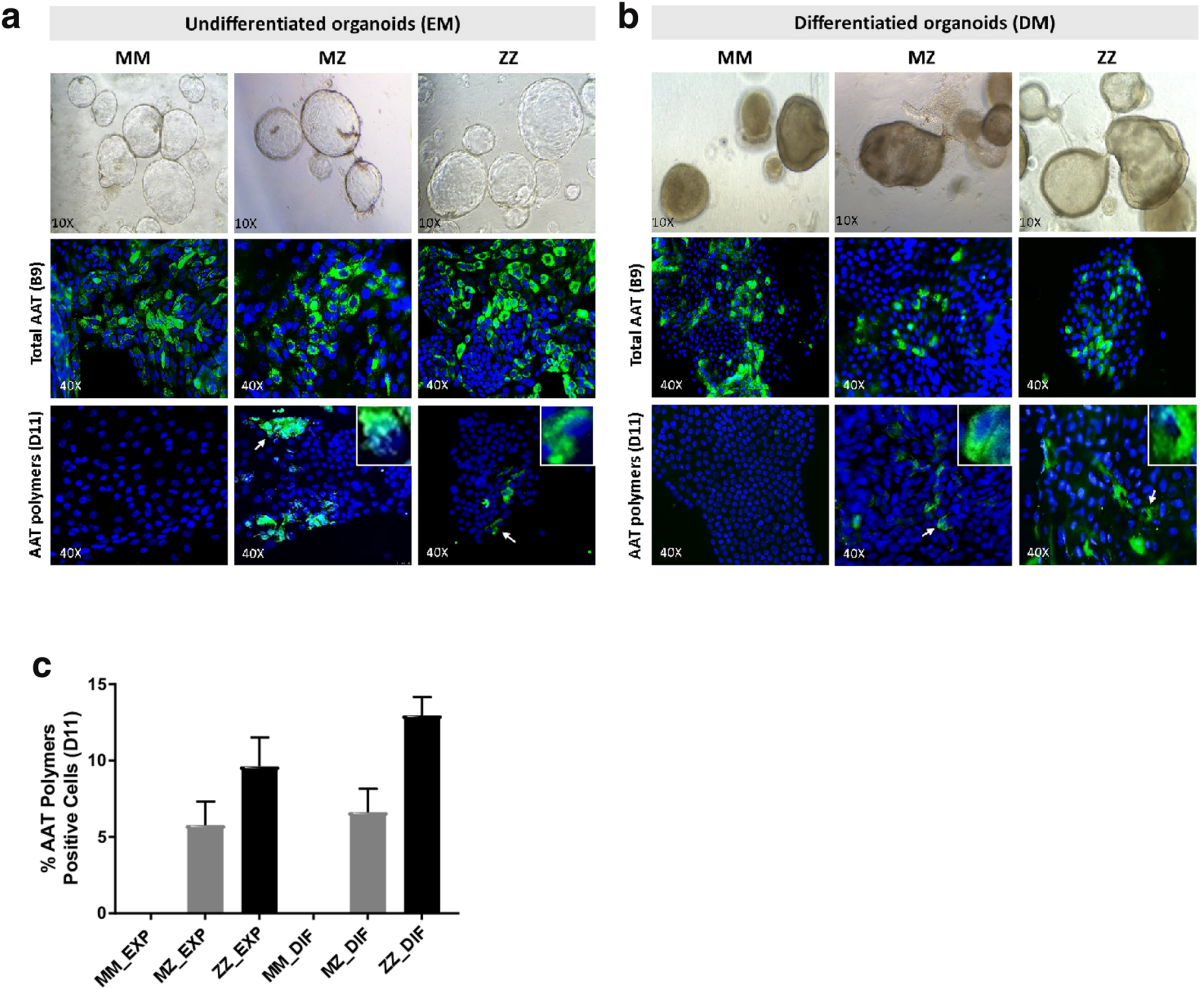
Immunofluorescence detection of AAT and AAT polymers. **a** Representative pictures of liver organoids in the expansion medium (EM) of MM, MZ and ZZ patients (images magnification ×10). **b** Differentiated liver organoids from MM, MZ and ZZ patients. Specific detection of total AAT protein with anti-AAT-B9 or with anti-AAT-D11 against AAT polymers, are shown in green fluorescence. Zoomed images of individual positive cells are showed in the right corner. **c** Quantification of AAT polymers (D11) positive cells in the different organoids MM, MZ and ZZ. Microscope Zeiss Ax10, camera Axio Cam Mrm Carl Zeiss and software AxioVision Rel.4.7 (images magnification ×40)

To detect specifically AAT protein, we performed immunofluorescence assays in undifferentiated and differentiated organoids from MM, MZ and ZZ AAT cases (Fig. 5). As expected, Z-AAT polymers were evident in Z, but not in M organoids. In both ZZ and MZ cases, AAT polymers were limited to restricted groups of cells or isolated cells. The quantification of cells positive for AAT polymer staining (D11 antibody) revealed that MZ organoids had around 5% of cells with polymer accumulation, approximately half of the positive cells found in ZZ organoids (10%) (Fig. 5c).

**Fig. 6:**
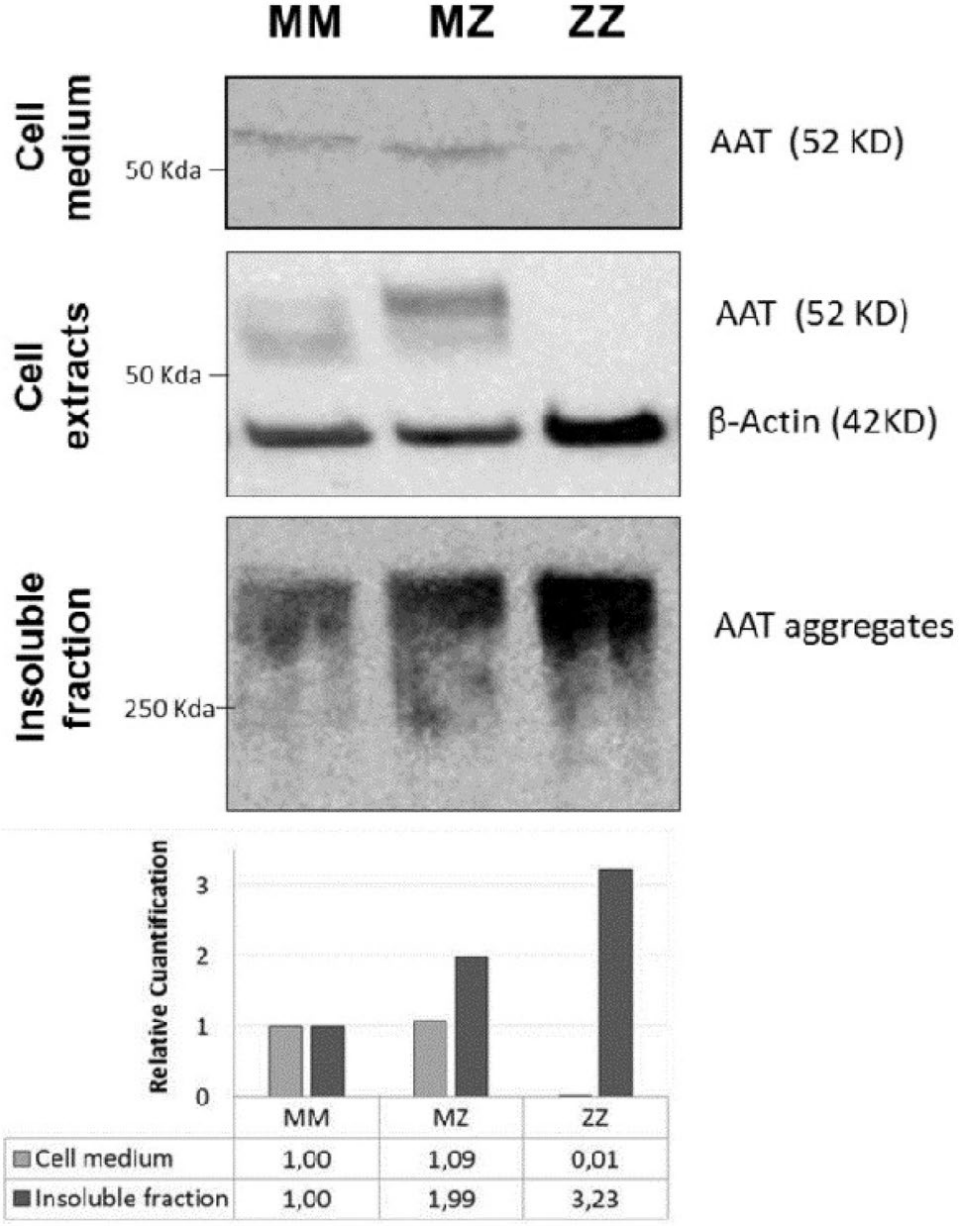
Representative western blot detection of AAT protein expression and secretion in MM, MZ and ZZ differentiated organoids. Blots of extracellular, cytoplasmic and insoluble fractions were incubated with anti-AAT-B9. The anti-β actin antibody was used as a control marker in the cytoplasm extract. Panels of figures were composed to show MM, MZ and ZZ AAT in the same order. Relative quantification with respect to MM amount of AAT in the extracellular medium and in the insoluble fraction of organoids is shown at the bottom

The western blot analyses revealed the presence of AAT protein in the extracellular medium and cell extracts of MM and MZ differentiated organoids (Fig. 6). The monomeric AAT protein was not detected in the cell extracts and extracellular medium of ZZ AAT organoids. By contrast, we found that the largest amount of insoluble AAT was present in ZZ compared to MZ or MM organoids (Fig. 6).

## Discussion

Inherited AAT deficiency is a common cause of genetic liver disease having a wide spectrum of presentations ranging from neonatal liver disease to an incidental finding of adulthood end-stage cirrhosis requiring transplantation. Currently, there are no biomarkers/predictors to identify AAT-deficient patients who will or not develop a severe course of liver disease. Moreover, the molecular mechanisms of AAT deficiency-related liver diseases remain largely unexplored.

Primary human hepatocytes reflect the metabolism and functionality of the human liver and are considered as the gold standard for hepatic ex vivo studies. However, the availability of primary human hepatocytes, especially from patients with rare inherited diseases such as AAT deficiency, is limited, and the function of isolated hepatocytes is hard to maintain ex vivo. When cultured in vitro, these cells undergo dedifferentiation, causing them to lose hepatocyte function. To solve the above issues, the development of new models allowing the culture and study of functional human hepatocytes is of great need [19–21].

To date, the 3D organoids are the best available representation of the liver cells with several advantages. First, organoids permit a long-term expansion with preserved genomic stability; second, organoid cultures recapitulate hepatocyte functions and allow to study molecular mechanisms of healthy and diseased liver, ex vivo; and third, organoids can be transplanted, as demonstrated in mouse models [7, 22], opening new avenues for regenerative medicine and gene therapy. These advantages of organoid systems together with emerging new technologies (such as 3D printing and imaging techniques) significantly contribute to the new strategies of how liver diseases are studied, and therapeutics developed.

Extensive analysis of cultured liver organoids revealed that the expanded cells preserve their genetic integrity over months in culture [7]. These results open up the avenue to start using human liver material expanded in vitro as an alternative hepatocyte source for studies of rare hereditary liver diseases. A variety of monogenic hereditary diseases affect the liver specifically, and these should be studied individually.

Therefore, in this study we established human liver organoid cultures reproducing the main characteristics of the wild-type M and pathogenic, deficient ZZ and MZ variants of AAT protein. When compared to 2D cell models [19], organoids are closer to liver tissue with respect to morphology, gene expression, and protein secretion. For example, the liver produces and secretes major proteins into the circulation, such as albumin, AAT, fibrinogens and apolipoproteins. Previous studies demonstrated that liver organoid cultures are able to express and secrete albumin and apolipoprotein B [7]. Patients with liver damage almost always have hypoalbuminemia caused by decreased albumin synthesis by the hepatocytes [23]. Data from a large cohort of patients revealed that ZZ AAT carriers have markedly reduced serum triglyceride and VLDL cholesterol concentrations compared to non-carriers [24]. In the model, we not only confirm that differentiated hepatocyte organoids express *ALB, SERPINA1, CYP3A4* and *APOB*, but also demonstrate that organoids from ZZ AAT individuals have lower expression of *ALB* and *APOB* in comparison to organoids from MM AAT. These findings further confirm that organoids from different genetic variants of AAT recapitulate specific features of the condition.

As an acute phase reactant protein, expression of AAT increases in response to various stimuli. Specifically, in the liver AAT is mainly regulated by IL-6-like cytokines including OSM. This process is mediated by the interaction between the hepatocyte promoter of *SERPINA1* and an OSM response element located at the 3′UTR of the AAT gene via the interaction of transcription factors like STAT3 [25]. We found that OSM significantly up-regulates AAT expression in MM and MZ organoids under undifferentiated and differentiated stages, whereas in ZZ organoids AAT was up-regulated only after differentiation. Currently, we are not able to explain these latter differences; however, the results provide clear evidence that organoids represent a good model to study the expression of liver AAT in relation to different AAT genotypes.

It is also important to point out that the expression of the *ST1C4* alternative short transcript of *SERPINA1* gene was up-regulated by OSM. Interestingly, some differences were observed between Z and M organoids. Namely, higher induction of the *ST1C4* was found in MM and MZ than in ZZ organoids after hepatic cell differentiation. The expression of *ST1C4* was previously found in the liver [5]; however, the transcriptional regulation and functional role of this transcript are not clear. The induction of *ST1C4* transcript in response to inflammatory signal further confirms its functional role and encourages further investigations. Hypothetically, this transcript can generate C-terminal peptide of AAT, which has been found to regulate bile acid synthesis in primary rat hepatocytes in vivo [26].

Based on clinical and experimental data, we have learned that hepatocytes from Z-AAT deficiency carriers can be damaged by gain-of-toxic mechanisms activated because of intracellular accumulation of Z-AAT protein [27]. As expected, the cultures of hepatic organoids derived from patients carrying Z-AAT mutation revealed intrahepatic aggregates of misfolded Z-AAT protein. The intracellular AAT accumulation (positive PAS-D staining) and polymerization (immunostainings with specific anti-Z-AAT polymer antibody) were abundant in organoids derived from ZZ and also detected in MZ subjects. Differently from MM and MZ organoid cultures, ZZ organoids showed almost no detectable secretion of AAT protein. These findings indicate that liver organoid cultures reflect AAT behavior seen in individuals with normal M- and mutant Z-AAT, and thus represent a valuable tool to study the molecular mechanisms underlying intracellular accumulation and secretion of AAT protein. Since organoids are derived directly from the affected individuals, they may also help us to elucidate the complex heterogeneity of liver disease among AATD carriers. In addition to Z-AAT mutation, many other rare alleles associated with AAT deficiency have been described [19, 28, 29] for which the potential damaging effects in hepatocytes are unknown. Organoids derived from patients carrying other than the Z variant would help to investigate the mechanisms of accumulation, secretion and degradation of these much less studied AAT variants.

Moreover, the development of organoid cultures has been successfully reported for other organs derived from the endoderm, such as the intestine, stomach, pancreas and lung, as well as for other internal organs derived from the mesoderm including the kidney, heart, cartilage, bone, reproductive organs and muscle, and also for tissues derived from ectoderm, both skin and its associated glands and neural ectoderm, giving rise to the brain, spinal cord and neural crest [8]. Organoids have proved very useful for a wide range of applications such as modeling human diseases, organogenesis and tissue development, drug screening, gene editing for monogenic diseases and regenerative medicine [8, 30].

Organoids represent an innovative approach to validate specific genetic alterations in the *SERPINA1* gene that are associated with liver disease development, and furthermore to identify and elucidate other genes associated with liver disease progression and to investigate putative treatments.

## Electronic supplementary material

Below is the link to the electronic supplementary material.

Supplementary material 1 (DOCX 58 kb)
