# Supplementary material for: Liver organoids reproduce alpha-1 antitrypsin deficiency-related liver disease
Source: Hepatol Int. 2019 Dec 13;14(1):127–37. doi: 10.1007/s12072-019-10007-y (PMC6994530; doi:10.1007/s12072-019-10007-y)
Supplement: Supplementary file 1 — Supplementary material 1 (DOCX 58 kb) [file 12072_2019_10007_MOESM1_ESM.docx]

**SUPPLEMENTARY MATERIAL (HEPATOLOGY INTERNATIONAL)**

**Liver Organoids reproduce Alpha-1 Antitrypsin Deficiency-related liver disease**

Gema Gómez-Mariano^1,*^, Nerea Matamala^1,*^, Selene Martínez^1^, Iago Justo^2^, Alberto Marcacuzco^2^, Carlos Jimenez^2^, Sara Monzón^3^, Isabel Cuesta^3^, Cristina Garfia^4^, María Teresa Martínez^5^, Meritxell Huch^6^, Ignacio Pérez de Castro^7^, Manuel Posada^8, 9^, Sabina Janciauskiene^10^, Beatriz Martínez-Delgado^1, 8^.

^1^ Molecular Genetics Unit, Institute of Rare Diseases Research. Institute of Health Carlos III (ISCIII), Madrid, Spain.

^2^ General and Digestive Surgery Department, Hospital Doce de Octubre, Madrid, Spain.

^3^ Bioinformatics Unit, Institute of Health Carlos III (ISCIII), Madrid, Spain.

^4^ Digestive Department. Hospital Doce de Octubre, Madrid, Spain.

^5^ Neumology Service. Hospital Doce de Octubre, Madrid, Spain.

^6^ Wellcome Trust–Medical Research Council Stem Cell Institute, University of Cambridge, Cambridge, UK.

^7^ Gene therapy Unit. Institute of Rare Diseases Research. Institute of Health Carlos III (ISCIII), Madrid, Spain.

^8^ Institute of Rare Diseases Research. Institute of Health Carlos III (ISCIII), Centre for Biomedical Network Research on Rare Diseases, CIBERER. Madrid, Spain.

^9^ Department of Respiratory Medicine, German Centre for Lung Research (DZL), Hannover Medical School, Hannover, Germany.

**Email authors:** Gema Gómez-Mariano^1,*^: [ggomezm@isciii.es](mailto:ggomezm@isciii.es); Nerea Matamala^1,*^: [neremat@hotmail.com](mailto:neremat@hotmail.com) Selene Martínez^1^: [selenemr29@gmail.com](mailto:selenemr29@gmail.com); Iago Justo^2^: [iagojusto@hotmail.com](mailto:iagojusto@hotmail.com); Alberto Marcacuzco^2^: [alejandro_mar@icloud.com](mailto:alejandro_mar@icloud.com); Carlos Jimenez^2^: [carlos.jimenez@inforboe.es](mailto:carlos.jimenez@inforboe.es); Sara Monzón^3^: [smonzon@isciii.es](mailto:smonzon@isciii.es); Isabel Cuesta^3^: [isabel.cuesta@isciii.es](mailto:isabel.cuesta@isciii.es); Cristina Garfia^4^: [cgarfia@hotmail.com](mailto:cgarfia@hotmail.com); María Teresa Martínez^5^: [mmartinezm.hdoc@salud.madrid.org](mailto:mmartinezm.hdoc@salud.madrid.org); Meritxell Huch^6^: [mh771@cam.ac.uk](mailto:mh771@cam.ac.uk); Ignacio Pérez de Castro^7^: [iperez@isciii.es](mailto:iperez@isciii.es); Manuel Posada^8^: [mposada@isciii.es](mailto:mposada@isciii.es); Sabina Janciauskiene^9^:; [sabinajanciauskiene@gmail.com](mailto:sabinajanciauskiene@gmail.com); Beatriz Martínez-Delgado^1,8^: [bmartinezd@isciii.es](mailto:bmartinezd@isciii.es)

**CORRESPONDING AUTHOR:** Beatriz Martinez-Delgado. Molecular Genetics. Instituto de Investigación en Enfermedades Raras (IIER). INSTITUTO DE SALUD CARLOS III. Ctra. Majadahonda-Pozuelo Km2,200. 28220 Madrid. Spain. Tel:+34918223152, Fax:+3918223269. Email: [bmartinezd@isciii.es](mailto:bmartinezd@isciii.es)

**Contents: Supplementary Figure; Supplementary Table 1; Supplementary Table 2; Supplementary Table 3.**

**
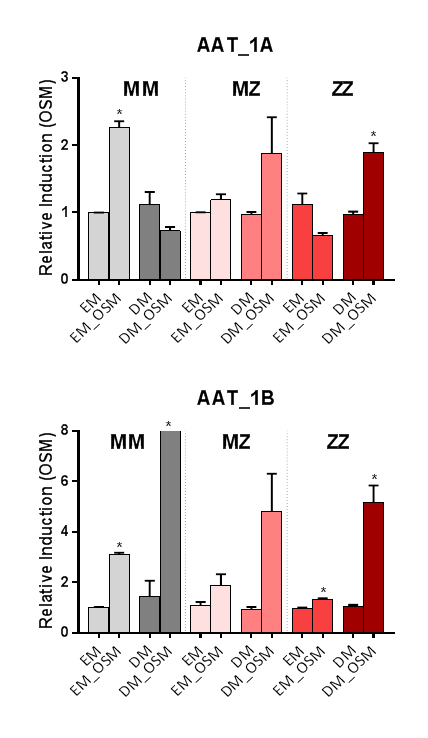
**

**Supplementary Figure.** Induction of *SERPINA1* gene transcripts 1A and 1B by exogenous stimulations with OSM (Oncostatin M). Relative gene expression of the analyzed transcripts comparing to untreated cells was measured both in organoids grown in expansion medium (EM) and in differentiated organoids (DM). Gene expression in organoids of liver biopsies of patients with normal (MM), heterozygous (MZ) and homozygous (ZZ) genotypes of AAT. Statistically significant values are shown by * (p <0.05).

**Table 1: Specific primers for QT-PCR amplification of genes LGR5, KRT19, ALB, APOB and CYP3A4. TaqMan probes Universal probe library, UPL, (Roche).**

| GENE NAME *†* | Forward Primer (5´-3´) | Reverse Primer (5´-3´) | Universal Probe Library for Human (Roche) |
| --- | --- | --- | --- |
| LGR5 | accagactatgcctttggaaac | ttcccagggagtggattctat | #78, cat.no. 04689011001 |
| KRT19 | gccactactacacgaccatcc | caaacttggttcggaagtcat | #71, cat.no. 04688945001 |
| ALB | agaggtctcaagaaacctaggaaa | ggttcaggaccacggataga | #44, cat.no. 04688040001 |
| APOB | acagctgattgaggtgtcca | agccactggaggatgtgagt | #90, cat.no. 04689151001 |
| CYP3A4 | aagaaactgagtcccacaaagc | ccagcaaaaataaagataattgattg | #50, cat.no. 04688112001 |

*†, GENE NAME: LGR5 (Leucine-rich repeat containing G protein-coupled receptor 5), KRT19 (Keratin 19), ALB (Albumin), APOB (Apolipoprotein B) and CYP3A4 (Cytochrome P450 family 3 subfamily A member 4)*

**Table 2: List of the top DEGs up-regulated after differentiation of liver organoids.**

| GENE_ID | NAME_GENE | Average Expression (EM) † | Average Expression (DM) ‡ | log2 (fold_change) | p_value | q_value |
| --- | --- | --- | --- | --- | --- | --- |
| SULT1C3 | Sulfotransferase Family 1C Member 3 | 0,34 | 90,62 | -8,08 | 5,00E-05 | 0,00061 |
| SULT1C2 | Sulfotransferase Family 1C Member 2 | 0,23 | 33,48 | -7,19 | 5,00E-05 | 0,00061 |
| ALB | Albumin | 0,18 | 22,83 | -6,98 | 5,00E-05 | 0,00061 |
| COL6A2 | Collagen Type VI Alpha 2 Chain | 0,04 | 4,71 | -6,98 | 0,0001 | 0,00113 |
| CD36 | CD36 Molecule | 0,65 | 80,46 | -6,95 | 5,00E-05 | 0,00061 |
| UGT2B11 | UDP Glucuronosyltransferase Family 2 Member B11 | 19,58 | 2302,24 | -6,88 | 5,00E-05 | 0,00061 |
| UGT2B28 | UDP Glucuronosyltransferase Family 2 Member B28 | 1,38 | 149,13 | -6,76 | 5,00E-05 | 0,00061 |
| ACMSD | Aminocarboxymuconate Semialdehyde Decarboxylase | 0,39 | 33,89 | -6,43 | 0,0041 | 0,02269 |
| SLC26A3 | Solute Carrier Family 26 Member 3 | 0,32 | 25,92 | -6,32 | 5,00E-05 | 0,00061 |
| CYP2C8 | Cytochrome P450 Family 2 Subfamily C Member 8 | 0,37 | 26,82 | -6,17 | 5,00E-05 | 0,00061 |
| HSD11B2 | Hydroxysteroid 11-Beta Dehydrogenase 2 | 2,44 | 173,27 | -6,15 | 5,00E-05 | 0,00061 |
| GDPD2 | Glycerophosphodiester Phosphodiesterase Domain Containing 2 | 0,37 | 22,88 | -5,96 | 5,00E-05 | 0,00061 |
| DAPK2 | Death Associated Protein Kinase 2 | 0,14 | 8,5 | -5,91 | 5,00E-05 | 0,00061 |
| APOB | Apolipoprotein B | 0,29 | 16,42 | -5,8 | 5,00E-05 | 0,00061 |
| HMGCS2 | 3-Hydroxy-3-Methylglutaryl-CoA Synthase 2 | 1,32 | 66,3 | -5,66 | 5,00E-05 | 0,00061 |
| MEP1A | Meprin A Subunit Alpha | 0,17 | 7,61 | -5,51 | 5,00E-05 | 0,00061 |
| SLC16A9 | Solute Carrier Family 16 Member 9 | 0,25 | 10,54 | -5,39 | 5,00E-05 | 0,00061 |
| RBPMS2 | RNA Binding Protein, MRNA Processing Factor 2 | 0,16 | 6,58 | -5,33 | 0,0001 | 0,00113 |
| AKAP12 | A-Kinase Anchoring Protein 12 | 0,22 | 8,09 | -5,18 | 5,00E-05 | 0,00061 |
| DISP2 | Dispatched RND Transporter Family Member 2 | 0,16 | 5,52 | -5,15 | 5,00E-05 | 0,00061 |
| ACE2 | Angiotensin I Converting Enzyme 2 | 2,08 | 69,75 | -5,07 | 5,00E-05 | 0,00061 |
| HRASLS2 | Phospholipase A And Acyltransferase 2 | 0,51 | 17,16 | -5,07 | 5,00E-05 | 0,00061 |
| ZNF114 | Zinc Finger Protein 114 | 0,08 | 2,61 | -5,06 | 5,00E-05 | 0,00061 |
| TMPRSS6 | Transmembrane Serine Protease 6 | 1,53 | 49,61 | -5,02 | 5,00E-05 | 0,00061 |
| FKBP5 | FKBP Prolyl Isomerase 5 | 3,87 | 123,39 | -4,99 | 5,00E-05 | 0,00061 |
| CYP3A4 | Cytochrome P450 Family 3 Subfamily A Member 4 | 3 | 88,03 | -4,88 | 5,00E-05 | 0,00061 |
| PHGR1 | Proline, Histidine and Glycine Rich 1 | 137 | 3482,4 | -4,67 | 5,00E-05 | 0,00061 |
| APOBEC1 | Apolipoprotein B MRNA Editing Enzyme Catalytic Subunit 1 | 1,34 | 33,59 | -4,65 | 5,00E-05 | 0,00061 |
| SAMD9 | Sterile Alpha Motif Domain Containing 9 | 2,73 | 21,66 | -2,99 | 5,00E-05 | 0,00061 |
| IGF2R | Insulin Like Growth Factor 2 Receptor | 12,88 | 25,67 | -0,99 | 0,0008 | 0,00630 |
| ACOT12 | Acyl-CoA Thioesterase 12 | 0 | 0,77 | - | 5,00E-05 | 0,00061 |
| APOC3 | Apolipoprotein C3 | 0 | 13,02 | - | 5,00E-05 | 0,00061 |
| AVP | Arginine Vasopressin | 0 | 1,08 | - | 5,00E-05 | 0,00061 |
| AWAT1 | Acyl-CoA Wax Alcohol Acyltransferase 1 | 0 | 0,51 | - | 5,00E-05 | 0,00061 |
| F13B | Coagulation Factor XIII B Chain | 0 | 0,76 | - | 5,00E-05 | 0,00061 |
| LINC01471 | Long Intergenic Non-Protein Coding RNA 1471 | 0 | 0,66 | - | 5,00E-05 | 0,00061 |
| SCNN1B | Sodium Channel Epithelial 1 Beta Subunit | 0 | 2,06 | - | 5,00E-05 | 0,00061 |
| SCNN1G | Sodium Channel Epithelial 1 Gamma Subunit | 0 | 0,56 | - | 5,00E-05 | 0,00061 |
| SPX | Spexin Hormone | 0 | 1,02 | - | 5,00E-05 | 0,00061 |
| TBPL2 | TATA-Box Binding Protein Like 2 | 0 | 1,05 | - | 5,00E-05 | 0,00061 |
| UGT2B4 | UDP Glucuronosyltransferase Family 2 Member B4 | 0 | 33,11 | - | 5,00E-05 | 0,00061 |
| UOX | Urate Oxidase (Pseudogene) | 0 | 0,7 | - | 5,00E-05 | 0,00061 |
| ZBTB16 | Zinc Finger and BTB Domain Containing 16 | 0 | 0,63 | - | 5,00E-05 | 0,00061 |
| KCNMB2 | Potassium Calcium-Activated Channel Subfamily M Regulatory Beta Subunit 2 | 0 | 0,74 | - | 0,00175 | 0,01156 |
| PLA2G12B | Phospholipase A2 Group XIIB | 0 | 0,67 | - | 0,00825 | 0,03904 |

*† EM: Expansion Medium; ‡ DM: Differentiation Medium.*

*p-value: The uncorrected p value of the test statistic; q-value: The FDR adjusted p value of the test statistic.*

**Table 3: List of the top DEGs down-regulated after differentiation of liver organoids.**

| GENE_ID | NAME_GENE | Average Expression (EM) † | Average Expression (DM) ‡ | log2 (fold_change) | p_value | q_value |
| --- | --- | --- | --- | --- | --- | --- |
| NUSAP1 | Nucleolar and Spindle Associated Protein 1 | 11,19 | 0,44 | 4,67 | 5,00E-05 | 0,00061 |
| CRIP1 | Cysteine Rich Protein 1 | 47,65 | 1,86 | 4,68 | 5,00E-05 | 0,00061 |
| ELOVL4 | ELOVL Fatty Acid Elongase 4 | 0,96 | 0,04 | 4,68 | 0,00165 | 0,01106 |
| KRT13 | Keratin 13 | 81,42 | 3,15 | 4,69 | 5,00E-05 | 0,00061 |
| FLNA | Filamin A | 20,51 | 0,78 | 4,73 | 5,00E-05 | 0,00061 |
| NR4A1 | Nuclear Receptor Subfamily 4 Group A Member 1 | 4,12 | 0,15 | 4,74 | 0,00155 | 0,01056 |
| ANLN | Anillin Actin Binding Protein | 7,46 | 0,27 | 4,77 | 0,00615 | 0,03114 |
| CCK | Cholecystokinin | 452,95 | 16,13 | 4,81 | 5,00E-05 | 0,00061 |
| GTSE1 | G2 And S-Phase Expressed 1 | 2,68 | 0,1 | 4,81 | 5,00E-05 | 0,00061 |
| TTK | TTK Protein Kinase | 4,12 | 0,14 | 4,85 | 0,0051 | 0,02684 |
| KLK6 | Kallikrein Related Peptidase 6 | 148,04 | 4,85 | 4,93 | 5,00E-05 | 0,00061 |
| ASF1B | Anti-Silencing Function 1B Histone Chaperone | 12,63 | 0,4 | 4,98 | 5,00E-05 | 0,00061 |
| CCNB2 | Cyclin B2 | 14,78 | 0,46 | 5 | 5,00E-05 | 0,00061 |
| NUF2 | NUF2 Component of NDC80 Kinetochore Complex | 4,25 | 0,13 | 5 | 0,0001 | 0,00113 |
| CSPG5 | Chondroitin Sulfate Proteoglycan 5 | 2,26 | 0,07 | 5,02 | 0,0096 | 0,04413 |
| CDC45 | Cell Division Cycle 45 | 5,77 | 0,18 | 5,03 | 5,00E-05 | 0,00061 |
| KCNH8 | Potassium Voltage-Gated Channel Subfamily H Member 8 | 0,81 | 0,02 | 5,29 | 0,0022 | 0,01388 |
| PHGDH | Phosphoglycerate Dehydrogenase | 23,04 | 0,58 | 5,32 | 5,00E-05 | 0,00061 |
| CEP55 | Centrosomal Protein 55 | 8,28 | 0,2 | 5,38 | 5,00E-05 | 0,00061 |
| GINS2 | GINS Complex Subunit 2 | 7,61 | 0,18 | 5,42 | 0,00015 | 0,00158 |
| UHRF1 | Ubiquitin Like with PHD And Ring Finger Domains 1 | 3,7 | 0,09 | 5,42 | 0,00015 | 0,00158 |
| MXRA5 | Matrix Remodeling Associated 5 | 2,43 | 0,05 | 5,5 | 5,00E-05 | 0,00061 |
| PTTG1 | PTTG1 Regulator of Sister Chromatid Separation, Securin | 42,37 | 0,85 | 5,64 | 5,00E-05 | 0,00061 |
| ASPM | Abnormal Spindle Microtubule Assembly | 2,09 | 0,04 | 5,66 | 0,00175 | 0,01156 |
| MEG3 | Maternally Expressed 3 | 22,5 | 0,43 | 5,69 | 0,0038 | 0,02146 |
| MYB | MYB Proto-Oncogene, Transcription Factor | 0,81 | 0,02 | 5,73 | 0,00325 | 0,01888 |
| CDC25C | Cell Division Cycle 25C | 2,11 | 0,04 | 5,76 | 0,00565 | 0,02912 |
| ALDH3A1 | Aldehyde Dehydrogenase 3 Family Member A1 | 326,17 | 5,82 | 5,81 | 5,00E-05 | 0,00061 |
| CDK1 | Cyclin Dependent Kinase 1 | 21,76 | 0,36 | 5,9 | 5,00E-05 | 0,00061 |
| EPHB3 | EPH Receptor B3 | 12,55 | 0,2 | 5,99 | 5,00E-05 | 0,00061 |
| SCARA3 | Scavenger Receptor Class A Member 3 | 8,42 | 0,11 | 6,23 | 5,00E-05 | 0,00061 |
| NEK2 | NIMA Related Kinase 2 | 5,34 | 0,06 | 6,47 | 0,0002 | 0,00200 |
| DTL | Denticleless E3 Ubiquitin Protein Ligase Homolog | 2,05 | 0,02 | 6,61 | 0,00925 | 0,04280 |
| BUB1 | BUB1 Mitotic Checkpoint Serine/Threonine Kinase | 4,81 | 0,05 | 6,62 | 5,00E-05 | 0,00061 |
| FAIM2 | Fas Apoptotic Inhibitory Molecule 2 | 2,45 | 0,02 | 6,67 | 0,00165 | 0,01106 |
| SHCBP1 | SHC Binding and Spindle Associated 1 | 3,46 | 0,03 | 6,68 | 5,00E-05 | 0,00061 |
| KCNE3 | Potassium Voltage-Gated Channel Subfamily E Regulatory Subunit 3 | 64,43 | 0,62 | 6,7 | 5,00E-05 | 0,00061 |
| CDC20 | Cell Division Cycle 20 | 27,53 | 0,21 | 7,04 | 0,0001 | 0,00113 |
| IL33 | Interleukin 33 | 10,26 | 0,07 | 7,15 | 0,0052 | 0,02727 |
| RRM2 | Ribonucleotide Reductase Regulatory Subunit M2 | 20,05 | 0,07 | 8,26 | 5,00E-05 | 0,00061 |
| BIRC5 | Baculoviral IAP Repeat Containing 5 | 11,98 | 0,04 | 8,27 | 5,00E-05 | 0,00061 |
| CLDN2 | Claudin 2 | 453,78 | 1,14 | 8,63 | 5,00E-05 | 0,00061 |
| SPINK4 | Serine Peptidase Inhibitor, Kazal Type 4 | 3892,87 | 6,64 | 9,2 | 5,00E-05 | 0,00061 |
| HIST2H2AC | Histone Cluster 2 H2A Family Member C | 0,89 | 0 | - | 0,0001 | 0,00113 |
| IGFL1 | IGF Like Family Member 1 | 0,52 | 0 | - | 5,00E-05 | 0,00061 |
| LOC150051 | Uncharacterized LOC150051 | 1,35 | 0 | - | 5,00E-05 | 0,00061 |
| LOC388282 | Uncharacterized LOC388282 | 0,53 | 0 | - | 5,00E-05 | 0,00061 |
| NUPR2 | Nuclear Protein 2, Transcriptional Regulator | 0,44 | 0 | - | 0,00035 | 0,00320 |
| RIBC2 | RIB43A Domain with Coiled-Coils 2 | 1,46 | 0 | - | 5,00E-05 | 0,00061 |
| SMPX | Small Muscle Protein X-Linked | 1,6 | 0 | - | 5,00E-05 | 0,00061 |
| SNORA25 | Small Nucleolar RNA, H/ACA Box 25 | 41,37 | 0 | - | 0,00015 | 0,00158 |

*† EM: Expansion Medium; ‡ DM: Differentiation Medium.*

*p-value: The uncorrected p value of the test statistic; q-value: The FDR adjusted p value of the test statistic.*
